# Three-Dimensional Printing Methods for Bioceramic-Based Scaffold Fabrication for Craniomaxillofacial Bone Tissue Engineering

**DOI:** 10.3390/jfb15030060

**Published:** 2024-03-01

**Authors:** Zeeshan Sheikh, Vasudev Vivekanand Nayak, Umer Daood, Anupreet Kaur, Hanan Moussa, Abbas Canteenwala, Pierre-Luc Michaud, Ísis de Fátima Balderrama, Edisa de Oliveira Sousa, Nick Tovar, Andrea Torroni, Michael Glogauer, Huzefa Talib, Paulo G. Coelho, Lukasz Witek

**Affiliations:** 1 Department of Applied Oral Sciences, Faculty of Dentistry, Dalhousie University, 5981 University Avenue, Halifax, NS B3H 1W2, Canada; 2 Department of Dental Clinical Sciences, Faculty of Dentistry, Dalhousie University, 5981 University Avenue, Halifax, NS B3H 1W2, Canada; 3 Biomedical Engineering, Faculty of Medicine, Dalhousie University, Dental Building, 5981 University Avenue, Halifax, NS B3H 3J5, Canada; 4 Department of Biochemistry and Molecular Biology, Miller School of Medicine, University of Miami, Miami, FL 33136, USA; 5 School of Dentistry, International Medical University, Jln Jalil Perkasa 19, Bukit Jalil, Kuala Lumpur 57000, Malaysia; 6 Faculty of Health Sciences, Schulich School of Medicine and Dentistry, University of Western Ontario, 1151 Richmond St., London, ON N6A 5C1, Canada; 7 Schulich School of Medicine and Dentistry, Western University, 1151 Richmond St, London, ON N6A 5C1, Canada; 8 Biomaterials Division, NYU Dentistry, New York, NY 10010, USA; 9 Department of Diagnosis and Surgery, School of Dentistry of Araraquara, Sao Paulo State University, R. Humaitá, Sao Paulo 14801-385, Brazil; 10 Department of Prosthodontics and Periodontology, Bauru School of Dentistry, University of Sao Paulo, Alameda Dr. Octávio Pinheiro Brisolla, Bauru 17012-901, Brazil; 11 Department of Oral and Maxillofacial Surgery, NYU Dentistry, New York, NY 10010, USA; 12 Hansjörg Wyss Department of Plastic Surgery, NYU Grossman School of Medicine, New York, NY 10016, USA; 13 Department of Dental Oncology, University Health Network, Princess Margaret Cancer Hospital, 610 University Avenue, Toronto, ON M5G 2M9, Canada; 14 Faculty of Dentistry, University of Toronto, 124 Edward St., Toronto, ON M5G 1X3, Canada; 15 DeWitt Daughtry Family Department of Plastic Surgery, Miller School of Medicine, University of Miami, Miami, FL 33136, USA; 16 Department of Biomedical Engineering, NYU Tandon School of Engineering, 6 MetroTech, Brooklyn, NY 11201, USA

**Keywords:** bioceramics, 3D-printing, bone tissue engineering, scaffold fabrication

## Abstract

Three-dimensional printing (3DP) technology has revolutionized the field of the use of bioceramics for maxillofacial and periodontal applications, offering unprecedented control over the shape, size, and structure of bioceramic implants. In addition, bioceramics have become attractive materials for these applications due to their biocompatibility, biostability, and favorable mechanical properties. However, despite their advantages, bioceramic implants are still associated with inferior biological performance issues after implantation, such as slow osseointegration, inadequate tissue response, and an increased risk of implant failure. To address these challenges, researchers have been developing strategies to improve the biological performance of 3D-printed bioceramic implants. The purpose of this review is to provide an overview of 3DP techniques and strategies for bioceramic materials designed for bone regeneration. The review also addresses the use and incorporation of active biomolecules in 3D-printed bioceramic constructs to stimulate bone regeneration. By controlling the surface roughness and chemical composition of the implant, the construct can be tailored to promote osseointegration and reduce the risk of adverse tissue reactions. Additionally, growth factors, such as bone morphogenic proteins (rhBMP-2) and pharmacologic agent (dipyridamole), can be incorporated to promote the growth of new bone tissue. Incorporating porosity into bioceramic constructs can improve bone tissue formation and the overall biological response of the implant. As such, employing surface modification, combining with other materials, and incorporating the 3DP workflow can lead to better patient healing outcomes.

## 1. Introduction

Autogenous bone grafts (autografts) are widely recognized as the preeminent standard for addressing bony defects [1]. These grafts, sourced from the patient, are acknowledged for their non-immunogenic nature and possession of osteoinductive and osteoconductive properties [2,3,4]. Nevertheless, the use of autografts is hampered by limited availability, necessitating a secondary surgical site for harvesting, thereby heightening the risk associated with inflammation, infection, and donor site morbidity [1,2]. Conversely, allografts consist of transplanted bone tissue obtained from the same species, typically harvested from cadaveric bone sources [2]. As a result, the utilization of allografts is constrained by the requirements for sterilization, processing, and the inherent potential risk of viral disease transmission, bacterial infection, or the prospect of host rejection [5]. Moreover, due to the requisite processing for sterilization, allografts exhibit diminished biocompatibility in comparison to autografts, resulting in an unpredictable osteoinductive potential [2,4,6].

A range of synthetic scaffolds has emerged as viable alternatives to autografts and allografts [2,6,7,8]. These porous constructs have a pivotal role within Bone Tissue Engineering (BTE) strategies, aiming to restore damaged or missing tissue. Optimal scaffolds are structures compatible with biological systems, acting as extracellular matrices (ECM), supporting cellular activity, and facilitating the growth of recently formed tissue [5,9]. The interconnected pores within scaffold structures facilitate nutrition, oxygen transport, cell migration, and tissue formation [10,11,12].

Among the material options available for scaffold fabrication, bioceramics have been explored for their biocompatibility and osteoconductivity. Certain bioceramics are sourced from biological origins, such as demineralized bone matrix [7], while others are artificially manufactured, including hydroxyapatite (HA), bioactive glasses, and β-tricalcium phosphate (β-TCP) [3,5,6,9]. Traditional methods for crafting bioceramic scaffolds involve techniques like salt leaching, freeze-drying, gas foaming, and the polymer template [13,14,15]. However, these methods present inherent challenges, including a lack of reproducibility and the absence of a structured, engineered internal pore network. This structural randomness resulting from these techniques has demonstrated implications for biological function, causing heterogeneity in the distribution of cells in vitro and non-uniform tissue ingrowth in vivo [13,16,17,18].

**Figure 1:**
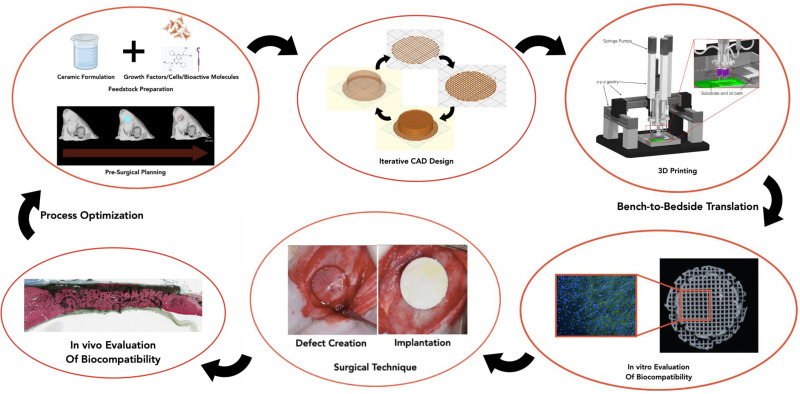
Schematic overview of the process workflow for 3DP in BTE Applications. Adapted from Refs. [25,26]. Adapted with permission from Ref. [27]. 2024 Wolters Kluwer Health, Inc. (Philadelphia, PA, USA) Ref. [28] 2024 John Wiley and Sons (Hoboken, NJ, USA) and Ref. [29] 2024 Wolters Kluwer Health, Inc.

Three-dimensional printing (3DP) technology in BTE (workflow schematic presented in Figure 1) is expected to play a crucial role in offering improved bone reconstruction, rehabilitation and regeneration [19,20]. Three-dimensional printing as a technique was initially introduced in 1986 by Charles W. Hull, known then as stereolithography [21]. Since then, various techniques have emerged with the aim of creating 3D constructions that replicate both the external and internal structure of the bone at the implanted site [22]. seeking to provide a vital framework for cell migration and adhesion, thereby initiating and strengthening the tissue regeneration process [23]. The use of 3DP in the craniofacial region has focused on rehabilitation of the defect site and restoration of facial and intraoral form and function, with the aim of preserving the existing bone and stimulating osteogenesis [20,24].

Three-dimensional printing technology in BTE has emerged with strong potential for fabricating patient-specific scaffolds for bone repair and regeneration applications. Over recent years, there has been an exponential growth in the research and application of 3DP techniques for BTE [30]. For instance, enhanced bone tissue regeneration using printed scaffolds has been achieved by tailoring them with surface modifications or incorporating bioactive factors, creating a favorable environment for tissue formation [31]. Moreover, incorporating growth factors has shown positive outcomes by promoting cell adhesion, proliferation, osteogenic differentiation, and bone formation [32,33]. 

The selection of bioceramic materials for creating scaffolds plays an extremely important part, as each material carries its own set of advantages and disadvantages. To achieve an optimal scaffold, it is imperative that it possess the ability to promote cell migration and proliferation, thereby stimulating the formation of bone tissue in the desired region. Furthermore, several other parameters related to the material can influence the quality of the printed structure. These factors encompass aspects such as the concentration, viscosity, and volume of the binding agent employed, the density and size of powder particles, the wettability between the powder and the binding agent, as well as the post-processing methods [34]. Furthermore, the scaffolds must also possess satisfactory mechanical properties. Therefore, detailed knowledge of each technique and material to be used is essential for proper planning and execution of the 3DP process. This review concentrates on the principal steps involved in producing 3DP scaffolds, various 3DP techniques, major bioceramic materials, and vital biological molecules used in BTE.

## 2. Bioceramics

Ceramics constitute a class of materials comprising inorganic and non-metallic solid components [32]. Upon exposure to high temperatures, these materials undergo a structural transition, resulting in bone-like arrangements due to the development of ionic and covalent bonds. Bioceramics, a subclass within this category, have gained significant attention for their potential in fabricating resorbable and implantable devices [35]. This increased interest is attributable to their ready availability, biocompatibility, bioactivity, hydrophilicity, stoichiometric similarity to natural bone, and osteoconductivity [36].

Bioceramics intended for BTE applications can be categorized as natural or synthetic. Natural bioceramics are sourced from human, bovine, porcine and piscine origins [3,6,32]. Conversely, synthetic alloplastic ceramics have been developed in laboratory settings through advancements in materials science research for BTE purposes. Synthetic bioceramics exhibit promising biological responses and offer a microenvironment comparable to natural bone [37]. For instance, the stoichiometry of tricalcium phosphate (TCP) and HA ceramics closely resembles that of amorphous bone precursors and bone minerals [38].

Despite the numerous favorable attributes associated with bioceramics, their inherent stiffness and low flexibility render them brittle, posing a challenge when shaping them into constructs [39]. Consequently, they exhibit inferior mechanical strength [40] and fracture toughness [41] compared to metallic materials, which limits their applications to anatomic areas that are not load-bearing. A detailed overview of bioceramics utilized in BTE is presented in Table 1.

### 2.1. Commonly Used Bioceramics

Hydroxyapatite (HA; Ca_10_(PO_4_)_6_(OH)_2_) is a calcium phosphate-based bioceramic with a calcium-to-phosphorus ratio between 1.50 and 1.67 [58]. HA is a major component of natural bone and constitutes ~65% of bone mass and the bulk of the inorganic components in bone tissue [8,59,60]. HA in bulk form demonstrates slow resorption in vivo, with a rate of <1% per year, is weak under tensile and shear forces, but has good compressive strength [61]. HA can be prepared using different methods depending on whether it is naturally harvested and synthesized in the laboratory. Naturally sourced hydroxyapatite can come from porcine [62], bovine [63], and piscine (scales) [64] sources and be converted into osteoconductive scaffolds, facilitating bone cell growth [65]. Synthetic HA transforms into a highly crystalline calcium phosphate when subjected to high temperatures (>1000 °C). The mechanical properties of HA scaffolds have been optimized by heat treatment and by varying the amount of ceramic material used for fabricating both load-bearing and non-load-bearing scaffolds for bone repair and regeneration [66].

The use of CaP-based ceramics, particularly HA, is based on their resemblance to the mineral phase found in bone [8,59,60]. When applied as a coating to titanium dental implants, it promotes bone-implant contact and fixation due to its chemical similarity to bone mineral and its ability to bond with bone [67]. The plasma-sprayed HA coating technique demonstrate excellent biocompatibility when used to coat implants [68] and demonstrate the ability to regulate osteoclast activity [69]. CaP materials exhibit a constraint resulting from weak chemical bonds when in contact with metallic surfaces [70]. Nevertheless, their high crystallinity, morphology, roughness, and wettability enhance their cell adhesion characteristics [71].

Tricalcium phosphate (TCP; Ca_3_(PO_4_)_2_) is another such bioceramic that has been extensively investigated for use as a bone substitute, as it is known to induce osteoblastic development of progenitor cells [47]. TCP is well known for its biocompatibility, bioactivity, osteoconductivity, and resorbability [61,72]. TCP is produced in situ by the dissolution precipitation process at 37 °C [47,73] and has two primary crystallographic forms, namely α-TCP and β-TCP [16], with the latter exhibiting a more favorable biological response and osteoconductivity [47,73]. To elaborate, β-TCP is a porous, osteoconductive ceramic that is slow to resorb [74]. However, studies have demonstrated β-TCP to have a more rapid resorption rate when compared to HA, maintaining osteoconductive properties—features that are desirable for achieving rapid replacement of scaffold with bone [74,75]. In addition, β-TCP has shown promise for use in BTE due to its mechanical strength and chemical stability. Its main mechanism of bioactivity is the partial dissolution and release of calcium and phosphate ion products, forming a biological apatite precipitate on the scaffold surface [76]. A previous study utilizing pH-controlled simulated body fluid reported that β-TCP begins to dissolve below pH 6.0 at 37 °C [77]. β-TCP constructs are usually sintered to high temperatures (~1100 °C) to facilitate densification and to render adequate mechanical strength (compressive strength: 2.5–16 MPa), making them an appropriate choice for low or medium load-bearing applications [47].

### 2.2. Other Ceramic Materials

Silicon carbide (SiC) bioceramics have been investigated for use as biomedical implants due to their bio-inertness, biocompatibility, ease of handling, capacity to be molded into any desired shape and physicochemical stability [78]. Research has shown that SiC has been extensively used for hip implants and load-bearing anatomical locations as it demonstrates high compressive strength, slow degradation and high biocompatibility [48,79]. SiC-based bioceramics constructs are typically sintered at high temperatures (between 1860 and 1950 °C) that yield high elastic modulus, a lower frictional coefficient, increased hardness, and chemical inertness [80]. Furthermore, SiC also demonstrates high wear and chemical resistance and low thermal expansion [81,82]. However, there are some notable disadvantages associated with SiC for bone tissue regeneration. First, silicon carbide is not naturally bioactive and lacks the inherent ability to promote osteogenesis, or bone tissue growth [81]. This could potentially hinder the speed and efficiency of the bone regeneration process compared to other bioceramics. Additionally, the cost of producing SiC can be relatively high [83], potentially limiting its accessibility to certain patients and healthcare institutions. Overcoming these challenges through ongoing research and development efforts is crucial to fully unlocking the potential of SiC in the field of regenerative medicine. 

Zirconium oxide (ZrO_2_) has been extensively studied for biomedical applications due to its biocompatibility, chemical stability, and excellent mechanical properties [84,85]. Zirconia can exist in three different crystallographic phases that are dependent on temperature, namely monoclinic (up to 1170 °C), tetragonal (up to 2370 °C), and cubic (above 2370 °C) [86]. In its tetragonal phase, it offers the most advantageous mechanical properties, and therefore, dopants can be employed to stabilize this phase at room temperature. Among the dopants used, yttrium oxide (Y_2_O_3_) is the most common, resulting in the material known as yttria-stabilized tetragonal zirconia polycrystals (Y-TZP) [87]. Previous research indicates that the flexural strength of Y-TZP can surpass 1000 MPa, while its fracture toughness can achieve levels of up to 10 MPa [88,89]. This outstanding mechanical performance can be attributed to a transformation toughening mechanism, in which tetragonal grains undergo a conversion to monoclinic (t-m) when the material experiences tensile stresses. This t m transformation leads to a 4% volumetric expansion, hampering crack propagation and thereby enhancing the ceramic’s resistance to damage [86,87,88,89]. 

These characteristics render zirconia a promising choice for applications in bone reconstructions. With a high melting point (2715 °C) and a sintering temperature range spanning from 1000 to 1450 °C, ZrO_2_ has been explored in the literature as a material of choice for 3DP scaffolds for bone tissue [87,90,91,92,93,94]. Nevertheless, the utilization of ZrO_2_ as BTE scaffolds can present some challenges, including, but not limited to, its non-resorbable characteristics [95]. Furthermore, the constraints of a non-reactive surface, low flexibility, and absence of osteoinductive properties pose challenges for its use in BTE. Consequently, researchers have prioritized the investigation of efficient techniques for altering the surface of ZrO_2_ scaffolds and surfaces. These methods include sandblasting, acid etching, atomic layer deposition, calcium phosphorus deposition, and laser treatment [96,97,98,99,100]. Sandblasting and acid etching procedures have been demonstrated to enhance the osteogenic characteristics and osseointegration capability of zirconia implants [96,101,102].

Barium titanate (BaTiO_3_) is another bioceramic used in BTE. It induces the piezoelectric effect, which is important for bone formation by maintaining a charged surface, thereby improving cell adhesion and proliferation [103,104]. The presence of a charged surface has been shown to enhance protein adsorption and improve cellular growth and metabolic function [105]. Scaffolds fabricated using BaTiO_3_ are effective for BTE due to their cytocompatibility and osteogenic differentiation, with high compressive strength and Young’s modulus [106,107,108]. Blending BaTiO_3_ with other bioactive materials like HA has further allowed for enhancement of bioactivity and implant stability by integrating with host bone tissues [109]. Despite its potential, there are some notable disadvantages, such as ensuring its long-term biocompatibility and safety for implantation in the human body, which is an ongoing area of research. Moreover, the technology for synthesizing BaTiO_3_ is still in its early stages, requiring further development and refinement to optimize its use for BTE. 

Silicate bioceramics have been applied in BTE due to their favorable characteristics for bone regeneration [110]. Calcium silicate is a fast-degradable bioceramic that releases ions (Si^4+^ and Ca^2+^) with bioactive properties to induce mineralization, angiogenesis, and osteogenesis [55,56]. Calcium silicate induces an increased pH and demonstrates an adverse effect on the behavior of cells because of its alkalinity [111]. However, the calcination temperature has a crucial role in determining their ion release and the behavior of cells on their surface [55]. Nanofibers of calcium silicate, subjected to a temperature of 1000 °C, have demonstrated the highest strength in inducing the osteogenic differentiation of bone marrow mesenchymal stem cells (BMSCs) [55]. However, in order to further enhance mechanical stability and compressive strength, calcium silicate nanofibers have been fabricated into three-dimensional microporous scaffolds and coated with gelatin [112]. Calcium silicate-based ceramic enhances the attachment and proliferation of osteoblasts, promoting bone ingrowth [57]. Additionally, calcium silicate fillers can be produced using the electrospray deposition technique on titanium [113], enabling a robust bond between the implant and the surrounding bone tissue for clinical use. In addition, surface modification of titanium with silica has been shown to enhance the mineralization behavior of osteoblast-like cells, as indicated by an increase in mineral production [114]. Moreover, calcium silicate biomaterials have been combined with natural polymers and collagen peptides due to their gradual resorption properties and the subsequent formation of apatite. In the literature, it has been reported that combinations such as gelatin methacryloyl (GelMA)/alginate/tri-calcium silicate and natural polysaccharides (copolymers of sodium D-mannuronate and L-guluronate)/natural polypeptides (gelatin)/calcium silicate/dicalcium phosphate dihydrate can be used to create an appropriate micro-environment for the regeneration and healing of oral bone defects [115,116].

### 2.3. Polymer-Ceramic Composite Materials

Scaffolds made of inorganic materials like HA, β-TCP, or other bioceramics display remarkable biocompatibility but suffer from brittleness. One strategy to enhance the mechanical properties of these brittle scaffolds involves the application of polymer coatings. This ensures the filling of existing cracks within the bioceramic structure with a polymer phase. It is hypothesized that this polymer phase not only fills the cracks but also acts to bridge them during fractures, thus increasing the toughness of the bioceramic scaffold. In addition, the polymer phase could be utilized as carriers for drugs and other biomolecules, such as growth factors, which enhance the functionality and bioactivity of the scaffolds.

As mentioned in previous sections, calcium phosphates, including HA and β-TCP, play an important role in the development of scaffolds for BTE. Polymers such as those derived from lactic acid (LABPs) offer mechanisms for promoting healing and minimizing infections while including controlled delivery of growth factors, antibiotics, and surface/chemical modifications [117,118]. These properties are similar to those present in calcium phosphate-based ceramic materials. Due to this, LABPs have been used in combination with ceramics yielding mixtures such as Polycaprolactone/TCP, and Polylactic Acid/HA yielding enhanced biocompatibility and mechanical properties relative to their individual constituent materials [119,120]. 

Considering the compositional organization of the polymer-ceramic composites, polymer matrices and ceramic reinforcement particles would be ideal, as the low mechanical strength of the polymer phase would be compensated by the ceramic phase. Additionally, ceramic particles would promote self-healing in the polymer matrices [121,122]. Hence, where the nature of the purely ceramic or polymeric scaffolds may not completely satisfy all dimensional, mechanical, and biological requirements, composites have been utilized and proven to be effective alternatives (Table 2) [123]. The fundamental procedure for creating polymer-ceramic scaffolds with interconnected microstructures involves the infusion of a sintered or partially sintered bioceramic scaffold with a polymer. Typically, a biodegradable synthetic polymer is employed, taking inspiration from the composition of bone, which comprises approximately 60% inorganic material (hydroxyapatite) and the remainder organic material (collagen). As such, a defining characteristic of such materials affecting their improved mechanical and biological properties are the interfaces between their various phases.

As in the case of any composite material, binders enhance interface properties. Shuai et al. described such interfaces as small regions in which the chemical composition of the two phases in the composite material has a significant change, forms a bond (Vanderwaal’s forces, chemical bonds, mechanical interlocks or electrostatic attraction) with each other, and can play a role in transferring load [124,125,126]. Considering polymer-ceramic composites, the literature also indicates self-healing due to the interface formed between the various phases. Techniques to achieve better interface bonding comprised polymer-ceramic composites include coupling agent modification that is described in detail further in this section; surface treatment such as polymer grafting [127,128] that can initiate polymerization of the monomer on the surface of the ceramic particles; and esterification, whereby ester bonds can be formed through a reaction between an acid and a hydroxyl group leading to the formation of an active group that can react with a biopolymer on the bioceramic surface [128,129]. Presently, coupling agent modification is the most frequently utilized method to improve interface bonding [124]. This operates by improving the degree of interface bonding between the two phases, imparting higher strength and superior properties when compared to the other techniques described above for use in load bearing BTE applications [130]. 

**Table 2:** A few polymer-ceramic composite materials used in BTE.

Polymer-Ceramic Composite	Improved Characteristics	Applications	Ref.
Poly(ε-caprolactone) (PCL)/ β-tricalcium phosphate (β-TCP)	Biocompatibility, mechanical properties, and antibacterial activity	Repair of bone defects	[131]
Poly(ε-caprolactone) (PCL)/ Hydroxyapatite (HA)	Hydrophilicity, cytocompatibility, mechanical behavior and elastic modulus	Repair of bone tissue	[131]
Poly(lactide-co-glycolide) (PLGA)/ Nano-hydroxyapatite (nano-HAP)	Mechanical properties	Repair of bone tissue	[132]
Poly(ε-caprolactone) (PCL)/ Hydroxyapatite (HA)/ Calcium sulphate (CaSO_4_)	Compressive strength of the scaffolds	Bone tissue engineered scaffolds	[133]

## 3. DP Methods for Fabrication of BTE Scaffolds

Investigations into granular bioceramic materials reveal that their random nucleation sites and the absence of spatial coordination pose obstacles to the organized directional growth of bone. This challenge is pivotal, as it impedes the complete restoration of both the form and function of recently regenerated bone tissue [94]. In addition, while conventional natural and synthetic bone grafting materials offer favorable healing outcomes and serve as supporting structures during bone formation, they have several disadvantages, including poor mechanical properties and poor resorption capabilities [3,5,7,8,9,134]. As such, the fabrication of complex geometries and interconnecting porous frameworks using bioceramics poses significant difficulties. Drawbacks associated with granular ceramics have spurred investigations into the creation of geometrically robust devices through 3D printing. Three-dimensional printing, alternatively referred to as additive manufacturing (AM), facilitates the incremental assembly of scaffolds based on bioceramics, forming elaborate and accurate constructs. The physical characteristics of the structures, encompassing factors like pore dimensions and configuration, the linkage between pores, and the overall geometry of the scaffold, can be specified through a three-dimensional model and produced by the machine [96,97]. The engineered 3D architectures achievable through 3DP enable the creation of scaffolds featuring interconnected pores and multiscale porosity, enhancing the integration between the scaffold and host tissue, facilitating the delivery of oxygen and nutrients to the scaffold’s core, and thereby fostering proper vascularization, cell proliferation, adhesion, differentiation, and overall bone tissue formation [135,136]. Consequently, the field of craniomaxillofacial (CMF) surgery has undergone a recent transformation, embracing individualized treatment approaches. The emergence of 3DP technology has been instrumental in the field of regenerative medicine, opening new avenues for creating customized scaffolds designed for site-specific defects while conserving space. Besides ensuring enhanced mechanical stability and preventing immediate failures, the incorporation of biological factors and/or bioactive molecules into these scaffolds further stimulates the promotion of osteogenesis and angiogenesis, endowing the structure with osteogenic, osteoinductive, and osteoconductive properties [135].

Advancements in Computer-Aided Design (CAD), Magnetic Resonance Imaging (MRI), and computed tomography (CT) have facilitated the reconstruction of regions of interest (ROIs). CT and MRI imaging enable the acquisition of a series of high-resolution images of the ROI, which can then be segmented for digital reconstruction. Subsequently, scaffold macro-geometric features are delineated to ensure adequate stabilization of the defect site and confer the necessary mechanical integrity at the site [137,138,139]. In subsequent stages, the reconstruction process involves converting the isolated ROI into a stereolithography (STL) file format to generate high-fidelity templates of the defect through discretization [140,141,142]. Upon determining the scaffold’s macro-geometric parameters, suitable slicing software is employed to slice objects, generating a multilayered, three-dimensional (3D) object composed of a sequence of parallel surfaces or planes with a specific interplanar spacing. Slicing converts the STL file into machine-level .gcode, containing essential coordinate-related instructions and auxiliary commands. These commands play a crucial role in customizing scaffold lattice parameters, such as pore spacing, layer height, and rod size, both prior to and during the printing process. The .gcode further details the scaffold’s printing orientation, offers virtual representations of the scaffold for ease of visualization, and allows print time optimization during rapid prototyping. Following this, scaffolds of varying sizes, shapes, and pore structures are manufactured to address the identified defect site through diverse 3DP methodologies. According to a pre-established standard (ISO/ASTM 52900:2021), additive manufacturing has been classified into seven different categories, namely binder jetting (BJT), directed energy deposition (DED), material extrusion (MEX), material jetting (MJT), powder bed fusion (PBF), sheet lamination (SHL), and vat polymerization (VPP) [143]. Of these categories, VPP and MEX workflows have been extensively studied to produce bioceramic scaffolds and will serve as the highlight of this review. Laser-assisted methods like selective laser sintering (SLS) and stereolithography (SLA), as well as light-assisted methods like digital light processing (DLP), are subclasses of VPP, while micro-extrusion techniques such as fused deposition modeling (FDM) and direct inkjet writing (DIW) are encompassed by the MEX workflow (Table 3) [28,75,137,144,145].

### 3.1. Vat Polymerization Techniques (VPP)

#### 3.1.1. Selective Laser Sintering (SLS)

**Figure 2:**
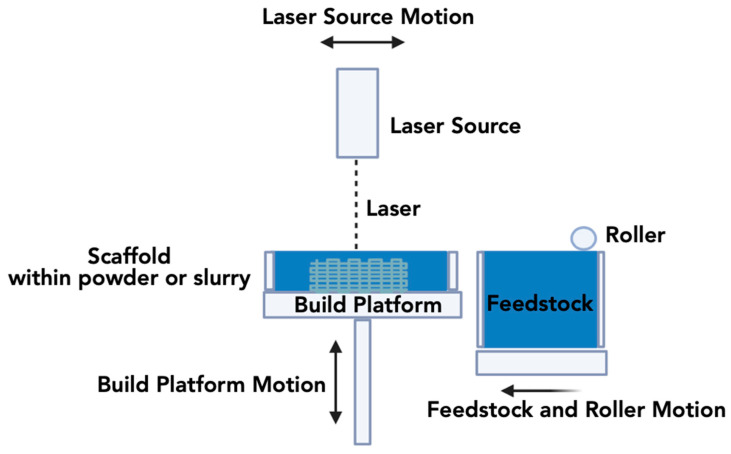
Schematic overview of the SLS methodology.

Introduced by Deckard and Beaman in 1986, SLS is a technique extensively utilized for the printing of custom implants, surgical planning guides, and constructs applied in orthopedics and dental tissue engineering [159,160]. In this method, a high-powered CO2 laser selectively and continuously fuses the surface of the powders, forming layers and resulting in the creation of the 3D construct [161]. Loose particles surrounding the sintered particles provide support, and with each layer scan, the powder bed descends as a roller spreads the subsequent layer of powder over the prior one. The application of SLS to ceramic-based materials can be achieved through either a direct or indirect method (Figure 2) [162,163]. The major concerns of utilizing SLS to 3D print ceramic-based tissue engineering devices are the presence of porosities and defects induced through shrinkage [164]. 

The direct SLS approach can be either slurry-based or powder-based. Slurry-based direct SLS has the advantage of starting from more homogenous and highly packed powder layers [163]. Thus, to ensure the dimensional accuracy of the structure, the powder or slurry is heated and sintered in situ [165]. However, the use of ceramics in the direct SLS method becomes challenging due to the need for high melting temperatures. Such high temperature requirements for ceramic-based materials and associated machine limitations to process such materials serve as disadvantages of this technique [166]. Nonetheless, an advantage is that these materials are more tolerant to temperature gradients [164]. 

Although the laser has the potential to reach the sintering temperature, achieving ideal densification of the ceramic powder within the brief laser exposure time is impractical. Moreover, extending the exposure time may lead to significant dimensional changes [166]. Additionally, factors such as laser energy consumption, extended cooling times, and laser scattering among ceramic particles can make this technique inefficient and costly to produce large and complex bioceramic scaffolds [34]. However, it is possible to enhance print quality by optimizing various factors, including particle size, particle shape, binder content, scanning speed, and laser energy [167].

On the other hand, the indirect SLS technique involves coating the chosen powder with a sacrificial organic polymer, which melts upon exposure to the laser, binding the ceramic particles together [162,168]. This indirect method comprises a three-step process: three-dimensional printing, thermal debinding and sintering [168]. However, the disadvantages of indirect ceramic-SLS are low resolution, poor surface finish, and porous microstructures within the fabricated parts [164]. While the indirect SLS technique allows the production of crack-free polymer-ceramic composite parts, there is a disadvantage associated with semi-crystalline polymers demonstrating between 4 and 5% of volume shrinkage upon solidification, which can cause component distortion [163]. Subsequent high-temperature sintering of the green constructs is then carried out to produce the final scaffold [163].

#### 3.1.2. Stereolithography Apparatus (SLA)

**Figure 3:**
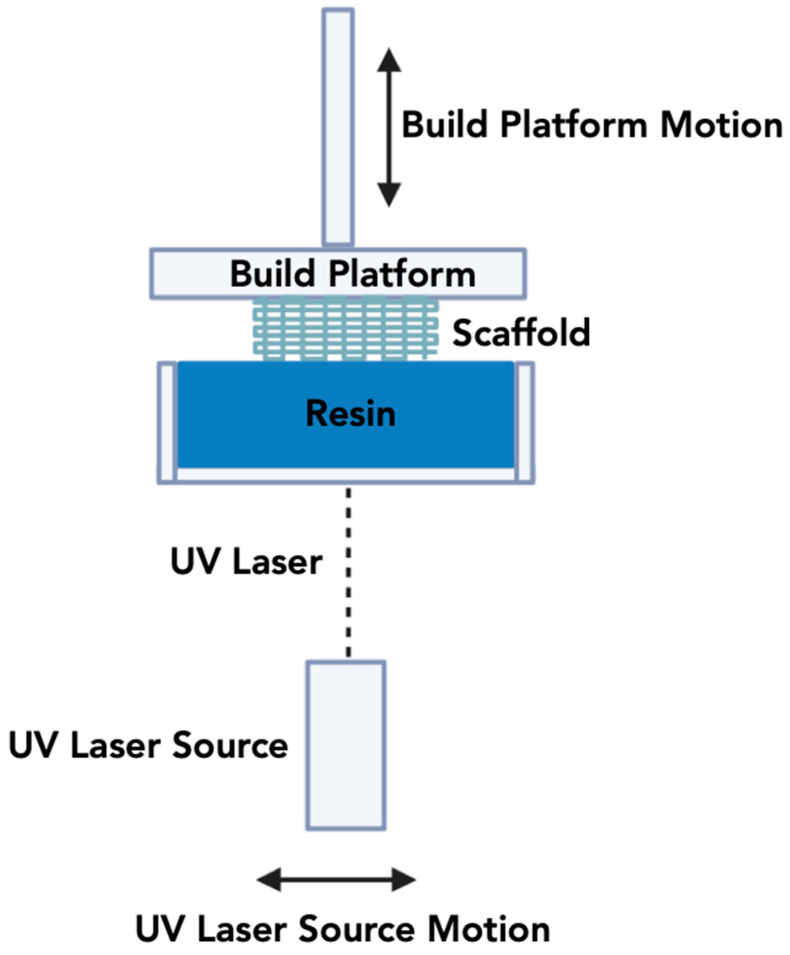
Schematic overview of the SLA methodology.

SLA, depicted in Figure 3, is a 3DP technique that allows the creation of highly precise and complex constructs with control over fine internal architectures (at the micrometer scale) and offers a high-quality surface topography [169,170]. Chuck Hull developed SLA in 1986 for manufacturing polymeric structures [21]. SLA involves a UV-curable photopolymer, a laser unit, galvanometric mirrors, support structures, an elevator, and a recoater blade [171,172]. The ultraviolet (UV) laser in SLA selectively crosslinks a photosensitive liquid resin in a layer-by-layer manner to construct a 3D object. Once a layer is completed, the resin bed moves upward and then descends back into the vat. This process continues until the final print is completed, achieving the intended scaffold [173]. The same principle can be applied to the use of SLA for printing ceramic materials. However, in this case, ceramic particles suspended in a slurry system replace the resin-based system with micro/nanometer-sized, light-sensitive monomers and a photo initiator that solidifies via photo-polymerization mechanism once exposed to a UV laser [174,175]. To obtain a smooth flow for printing and homogeneity in the print, the ceramic resin is required to have a long shelf life and appropriate rheological behavior [176].

SLA is used to fabricate scaffolds for bone regenerative applications using materials such as HA, β-TCP, alumina, ZrO_2_, and bioactive glasses [175]. A primary difference between the traditional SLA and ceramic SLA methods is the contribution of scattering phenomena due to the addition of ceramic particles relative to the light-sensitive monomer [174]. To elaborate, the ceramic particles scatter UV light, which reduces curing depth, resolution, and increases the printing time [177]. Hence, smaller particle sizes lower the occurrence of scattering and are hence preferable for the SLA technique, coupled with the fact that the cure depth can be controlled by adjusting the power of the laser, exposure time and scan speed [178,179]. Organic components have to be calcined to be removed and eventually sintered at high temperatures to fuse/densify the ceramic particles [180]. 

#### 3.1.3. Digital Light Processing (DLP)

**Figure 4:**
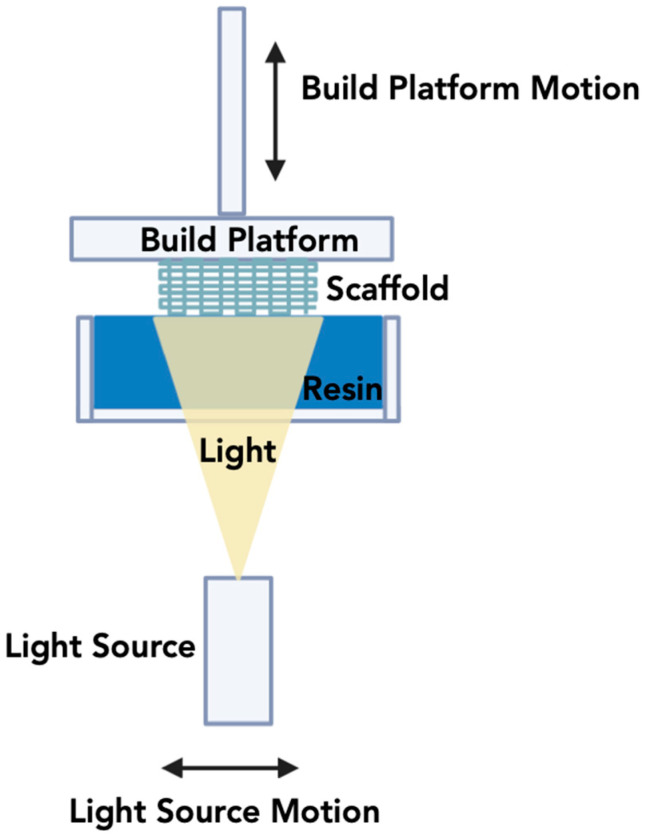
Schematic overview of the Digital Light Processing methodology.

Digital light processing (DLP) employs resins made of photopolymers to create three-dimensional structures under an illumination source [181,182]. The key components of this 3DP technique are a projector screen made up of pixels with digital light, a digital mirror device made of numerous micro-mirrors that navigate light from the projector, a conveyor and a resin tank that contains the feedstock (Figure 4) [157]. Speed and printing efficiency with great dimensional accuracy are the main advantages of using the digital light processing method [183]. Digital Light Processing (DLP) is an advanced AM technology that is increasingly being explored and adapted for use in BTE, with a focus on the fabrication of ceramic-based scaffolds [184,185,186,187]. This innovative process leverages a digital micromirror device (DMD) or a liquid crystal display (LCD) to precisely control and modulate light exposure patterns in a layer-by-layer fashion. In the context of BTE, DLP employs photosensitive ceramic resins as the starting material. These resins are formulated to include ceramic particles or precursors, which respond to photopolymerization upon exposure to ultraviolet (UV) light or other suitable light sources [183]. The DLP apparatus projects digital images, dictated by a computer-aided design (CAD) model, onto the ceramic resin. As the light interacts with the resin, it triggers the photopolymerization reaction, leading to the solidification of the material in the desired pattern. This highly controlled, layer-by-layer process enables the creation of intricate and precise ceramic scaffolds that closely mimic the structure and properties required for effective BTE [186].

The advantages of DLP for ceramic-based BTE are manifold. Foremost, it offers the capability for patient-specific customization, allowing the creation of implants that match an individual’s anatomical characteristics [188]. This personalization enhances the likelihood of implant integration, minimizes the risk of rejection, and promotes optimal bone healing. Moreover, DLP allows for meticulous control over the architecture of the scaffold, including the size, shape, and distribution of pores, which are critical factors influencing cell infiltration, nutrient diffusion, and vascularization within the scaffold. These parameters are vital for supporting bone tissue regeneration. Furthermore, DLP-printed ceramic structures typically demonstrate excellent mechanical properties, making them suitable for load-bearing applications in bone reconstruction [182]. However, post-processing steps like sintering may be required to enhance the mechanical strength and biocompatibility of the printed ceramic structures. As the field of DLP for ceramic BTE continues to advance, it holds immense potential for revolutionizing the field by delivering customized, high-quality ceramic scaffolds that foster efficient bone regeneration, leading to improved patient outcomes.

### 3.2. Material Extrusion Techniques (MEX)

#### 3.2.1. Direct Inkjet Writing (DIW)

**Figure 5:**
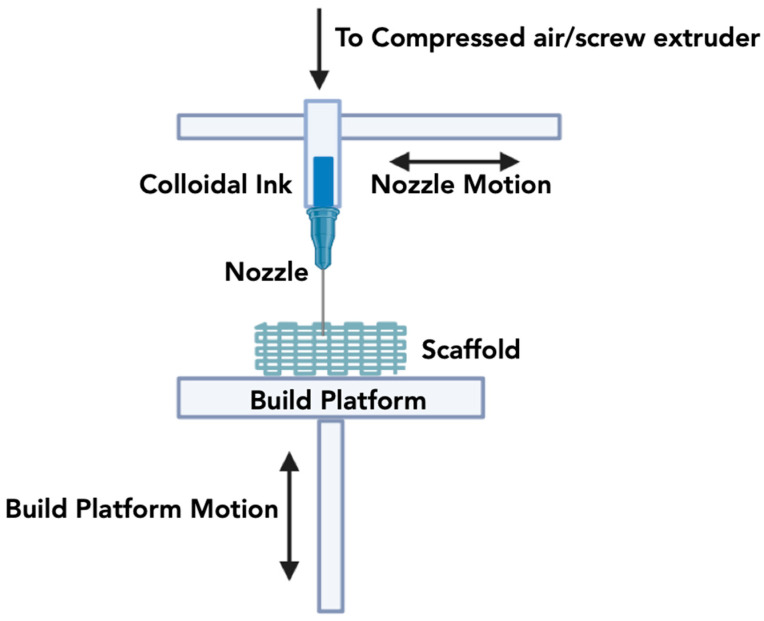
Schematic overview of the Direct Ink Writing methodology.

The DIW technique, showcased in Figure 5, originated with Cesarano in 1997 and is also commonly referred to as micro-robotic deposition or robocasting. This approach involves the layer-by-layer creation of objects by extruding and depositing a water-based colloidal suspension (referred to as ink), which comprises a substantial amount of ceramic powder (usually exceeding 40%), employing a movable nozzle managed by a robotic deposition arm or gantry [189,190,191]. In contrast to alternative methods, DIW presents superior speed and cost-efficiency, enabling the entire procedure, encompassing production, drying, and sintering, to conclude within a relatively short period, typically between 24 and 48 h. Compared to other techniques, DIW offers greater speed and cost-effectiveness, enabling the entire process, including fabrication, drying, and sintering, to be completed within a relatively short timeframe, typically ranging from 24 to 48 h [192].

A DIW printer achieves three-axis motion by inputting a tool path (.gcode) to a computer numerical controller. These printers generally include a fixed platform equipped with a mobile gantry. The controller governs the gantry’s movements and manages the downward motion of the syringe pumps containing the colloidal gels [27,193]. Multiple extrusion nozzles permit the simultaneous deposition of diverse colloidal gels, comprising the primary printing material (such as β-TCP) and fugitive support material (like Carbon Black) [28]. This support material is typically introduced during the printing process and subsequently eliminated through dissolution or melting once the printing operation is finalized [74]. The use of fugitive support material facilitates the creation of elements with internal voids or overhangs that would otherwise be challenging to produce without such support.

During the DIW procedure, the printer’s extruders adhere to the Cartesian coordinate pathway specified in the .gcode file while placing the gel or slurry onto a substrate [194]. The time required to build periodic structures, such as lattices or scaffolds, depends on various factors, including the nozzle diameter, extrusion rate from the nozzle, overall scaffold volume, and printing speed. Challenges linked with the DIW method involve recurrent nozzle clogging, sensitivity of ink to processing parameters, requirements for optimizing colloidal ink, and ‘filter pressing’ caused by constant ink extrusion pressure and particle separation from the liquid phase [195]. In addition, developing a colloidal gel (or ceramic ink) that is suitable for the deposition process with a shear-thinning property and yet is able to retain its shape after extrusion is a major challenge with the DIW technique [196,197]. As such, there is usually a high solid content, which prevents crack formation during the drying process and enables the deposited ink to retain its shape or volume after drying [198].

#### 3.2.2. Fused Deposition Modeling (FDM)

**Figure 6:**
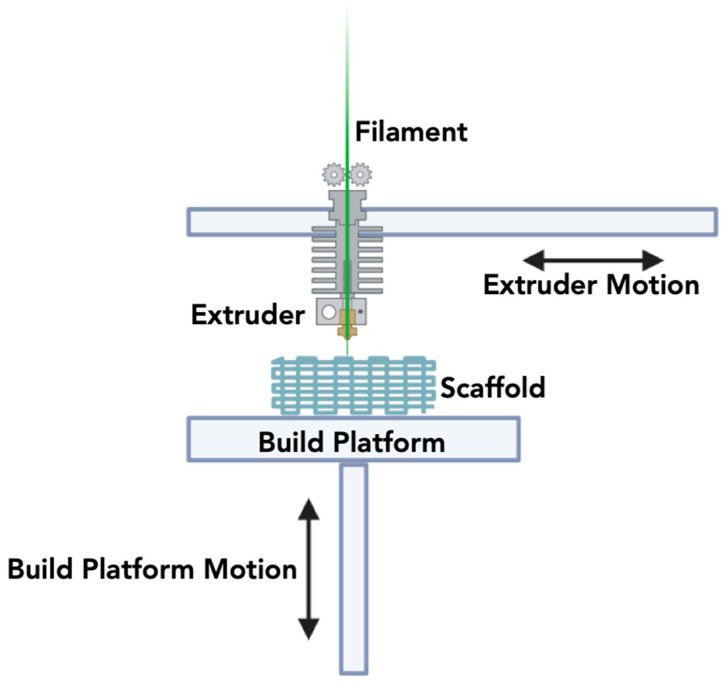
Schematic overview of the Fused Deposition Modeling methodology.

Thermoplastic polymer filaments are used to fabricate 3D constructs by fusion deposition modeling, an extrusion-based 3DP technique. FDM functions on the principle of AM, systematically depositing material in a layered manner onto the surface [199]. The essential components of the FDM process include material feeding, gantry, print head, and the forming surface [200]. In this printing method, thermoplastic filaments undergo a melting process between rollers and are subsequently extruded through a nozzle onto the construction surface (Figure 6). FDM offers the advantages of enabling multi-material printing, ensuring high design quality, and enhancing mechanical properties [201]. Although this method is conventionally linked with thermoplastic materials [147], its application has extended to ceramics, presenting new opportunities for crafting ceramic components with intricate geometries [202,203]. In FDM for ceramics, a ceramic-filled filament is extruded layer-by-layer, following a computer-aided design (CAD) model. Once the ceramic filament is deposited, it is heated to fuse the particles together, creating a solid structure [204]. This process enables the production of ceramic parts with intricate shapes that would be challenging to achieve through traditional ceramic forming methods. 

Despite its advantages, FDM for ceramics comes with some challenges and limitations. The high-temperature requirements for sintering or post-processing ceramic FDM parts can pose difficulties in terms of energy consumption and equipment costs [164]. Moreover, achieving high levels of detail and surface finish in ceramic FDM parts can be challenging, as the process may result in a rougher texture compared to conventionally manufactured ceramics. Generally, the limitations of processing ceramic materials with FDM demonstrate an inferior print resolution, poor surface finish, low density, and poor mechanical performance. In addition, the printed parts would, in some cases, require infiltration with isostatic pressing for greater quality improvements [164]. 

The technique’s primary benefit is its ability to enable rapid prototyping and on-demand manufacturing at low costs. However, the choice of ceramic materials available for FDM is limited compared to traditional ceramic processing techniques, or DIW. FDM is a slow process and can thereby hinder mass production relative to other techniques like DLP. As research and development in the field of ceramic FDM continue to advance, it is likely that many of these challenges will be addressed, further expanding the utility of this technology in various industries.

### 3.3. Process Optimization and Post Processing

The effective and precise application of 3D printing processes relies on an extensive optimization of various parameters. These parameters include the size of the powder, the density of the powder bed, the surface characteristics, and the properties of the binder, especially in micro-extrusion techniques. In resin-based printing, parameters involve laser power source attributes, resin optical characteristics, and the size of ceramic particles within the resin [181,205,206].

Some of the advantages of 3DP are its scalability to large sizes, relatively low cost, control over the pore geometry and pore size, and interconnectivity of constructs [136,207,208]. Nevertheless, a significant challenge of this technique lies in the extensive optimization required to create a construct with a precisely porous structure [34]. Optimizing the powder size plays a crucial role in enabling the smooth flow of particles in DIW and in achieving a tightly packed powder bed using the laser-assisted 3DP method. This optimization facilitates the creation of intricate details within the printed scaffold [209]. Generally, the use of finer powder particles enables more accurate and delicate microscale features, leading to a smoother surface finish in the printed constructs. In contrast, larger-sized particles spread more easily over the powder bed and allow for efficient binder penetration. However, utilizing excessively large particles with high flowability results in reduced stability and powder density in the printing bed. The roundness of the particles significantly enhances powder flowability during printing [210].

In the post-processing of bioceramic scaffolds, sintering is a critical step in the various 3D printing techniques detailed in this review. It serves to enhance the mechanical properties and eliminate the organic binder, burn off fugitive support material, remove undesirable polymeric impurities (employed in the ink formulation step in DIW), and/or fuse the individual ceramic particles together. Sintering results in both physical and chemical changes in the printed constructs, whereby high interconnectivity between particles is achieved, making the printed constructs stronger and more resilient to fracture by mechanical loading [34]. Although sintering is an essential step, it results in volumetric shrinkage [136], which can lead to dimensional inaccuracy. In addition, the shrinkage may not be uniform, leading to the incorporation of residual stresses and crack formation in the printed construct, which requires a good understanding of feedstock preparation, geometric dimensioning, and tolerance prior to ink, filament, or slurry (feedstock) preparation. To replace sintering, acid-based binders are now being utilized and explored and could serve as a viable option in some cases where sintering is not possible [205,211].

Sterilizing 3D-printed (3DP) devices is a critical aspect of their medical applicability. The primary sterilization methods include steam, ethylene oxide (EtO), or γ-radiation. Steam sterilization employs high temperatures (up to 135 °C, but generally between 121 and 135 °C) and is cost-effective and non-toxic. It exposes the devices to high-temperature steam under pressure for a calculated duration to eliminate microorganisms [212]. EtO operates at lower temperatures within the range of 37–63 °C. Ethylene oxide functions by alkylating proteins and DNA in microorganisms [213]. EtO sterilization is a longer process than steam sterilization and requires aeration to eliminate residue [214]. However, as EtO sterilization is performed at lower operating temperatures, it is compatible with a wider range of materials, especially those sensitive to heat or moisture. γ-radiation sterilizes through irradiation, typically at levels exceeding 25 kGy, and does not necessitate an extended aeration process [214]. When considering devices meant for implantation versus those used as intraoperative models for reference, distinct factors come into play. For 3DP devices designed for implantation, it is crucial that the sterilization process does not compromise the device’s structural and mechanical integrity. Consequently, it is essential to consider the ability of the material to withstand these different sterilization methods while also considering factors such as the availability of the aforementioned techniques.

### 3.4. Latest Technological Improvements and Applications

To fulfill the functional prerequisites of the 3DP technique for bone scaffolds, each step of the process plays a crucial role. The pre-processing stage begins with the acquisition of images of the defect site, which can be conducted through scanning the region via computerized tomography and magnetic resonance imaging [32]. The obtained data is then transferred to computer-aided design (CAD) software for the planning phase and design of the scaffold. Subsequently, the information is exported to the 3D printer. In this phase, parameters such as printing speed, layer thickness, alignment, printing temperature, and filling density are defined, depending on the chosen technique and material [215]. Considering this, emerging technologies such as machine learning and artificial intelligence (AI) are being applied to assist in the quality control of AM processes. Studies have highlighted the benefits of machine learning for optimizing 3DP techniques [216,217,218,219]. ML, a subfield of AI, primarily focuses on creating analytical models capable of identifying patterns in data and making predictions of future outcomes based on prior information [218]. ML has been shown to assist in determining ideal printing methods that result in faster printing of structures with higher shape fidelity and superior mechanical properties while also providing insights into the impact of different parameters on the printing process [219].

## 4. Biological Factors and Bioactive Molecules

With the emergence of tissue engineering methodologies, such as the utilization of 3D scaffolds that provide conducive environments for cell migration and proliferation, there has been a focus on enhancing the rate of bone formation and regeneration by incorporating exogenous osteogenic cells, especially through the application of stem cells (SCs) [139,220,221,222]. Therefore, a clear understanding of the selection of cell sources and the strategies employed to enhance osteogenic differentiation is essential. Osteoblasts possess strong osteogenic potential and can synthesize and secrete bone matrix, thereby promoting mineralization and bone formation. However, their main disadvantages include the low availability of donor sites, low proliferative capacity, and longer incubation times [139]. Consequently, stem cells (SCs) have been extensively investigated in BTE [139,223,224,225]. SCs are undifferentiated cells with the capacity for self-renewal, proliferation, and, with appropriate signaling, differentiation into different lineages of specialized cells. These cells can be categorized as embryonic stem cells (ESCs), induced pluripotent stem cells (iPSCs), and postnatal adult stem cells based on their source [139]. Considerable efforts have been made towards ESC differentiation into an osteogenic lineage for BTE; however, there is currently no consensus regarding their tumorigenicity, immunogenicity, and ethical or safety issues [139,225]. Similarly, with the use of iPSCs, although some attempts have been made towards their differentiation in osteogenic lineages, further study and improvements are needed for the optimization of induction approaches and control of cellular differentiation [139]. On the other hand, adult stem cells have been the most investigated in BTE research, including bone marrow mesenchymal stem cells (BMSCs), human periapical cyst mesenchymal stem cells (hPCy-MSCs), dental pulp stem cells (DPSCs), and adipose-derived stem cells (ASCs), among others. 

Adult stem cells have demonstrated a potential to differentiate into osteoblasts, with BMSCs specifically recognized for their ability to differentiate into osteoblasts, chondrocytes, or adipocytes [139,225]. Using autologous BMSCs has yielded optimal outcomes in repairing mandible defects, showcasing promising potential for bone regeneration in the CMF region [226]. Clinical data has also shown an enhanced rate of bone formation with BMSCs [38,39,40,41,42,43]. However, the procedure for aspirating BMSCs is invasive and painful for patients. Moreover, their retrieval is challenging due to the low frequency of BMSCs in human bone marrow (0.001%–0.01%) [227]. This scarcity worsens with age, further limiting the attainment of a significant osteogenic effect [225]. An additional category of dental stem cells, hPCy-MSCs, collected from pathological tissue, have been reported to exhibit MSC-like properties, such as plasticity, a high proliferation rate, and the potential to develop into osteoblasts, neurogenic-like cells, and adipocyte-like cells [228]. However, the utilization of hPCy-MSCs is limited due to the lack of evaluation of their immunomodulatory properties, and the results are predominantly derived from in vitro experiments [229]. Another source of stem cells is DPSCs, obtained from extracted third molars and premolars frequently removed for orthodontic reasons. They are more accessible than BMSCs and have been recognized as a cellular source for regenerative medicine [230]. Studies have demonstrated positive outcomes with the incorporation of DPSCs in the context of BTE [231]. However, there are cases where stem cells fail to be recruited or lack adherence to the scaffolds. In these situations, a notable challenge emerges due to the difficulty in securing a sufficient cell population, consequently jeopardizing the potential for top-tier tissue regeneration [232].

Therefore, the proposal for in vitro culture expansion aims to acquire an adequate quantity of cells for clinical application. Nevertheless, cell expansion needs to be carried out in facilities that follow good laboratory practices, which are laborious, expensive, and time-consuming. Moreover, issues such as loss of proliferative and differentiation capability during cell expansion, along with heightened risks of pathogen contamination and genetic transformation, are associated with these expansion procedures [225]. Considering this, numerous strategies and methods have been developed to enable clinicians to utilize growth factors, such as platelet concentrates (platelet-rich plasma—PRP and platelet-rich fibrin—PRF), fibroblast growth factor (FGF), vascular endothelial growth factor (VEGF), bone morphogenetic protein (BMP), platelet-derived growth factor (PDGF), and dipyridamole (DIPY), for regenerative purposes [220,221,233]. Growth factors transmit signals to target cells through receptor binding, leading to the activation of specific genes. Therefore, a site-specific dosage relationship and sequence of biomolecules determine the cellular response as well as the quality and quantity of tissue regeneration [234]. However, the multiplicity of applied factors, carriers, and methods utilized in the literature makes it challenging to evaluate the most predictable therapy [235]. 

The most widely used osseoinductive growth factor is BMP, which is a member of the transforming growth factor superfamily—β, isolated and purified from bone extracts [223]. BMP displays chemoattraction toward osteoprogenitor cells and SCs, promoting their proliferation and differentiation into mature osteoblasts. It also up-regulates VEGF to enhance angiogenesis [236]. In large bone defects, BMP-2 delivered locally via scaffolds has been shown to result in increased osteogenesis compared to BTE devices (scaffolds) alone [237,238,239]. BMP-2 application has resulted in an increase in the both quantity of bone formation and its quality, with an increase in the rate of bone-forming markers such as osteocalcin [235]. Reported side effects of using BMPs include severe inflammation, ectopic bone formation, and premature suture fusion [138]. An additional biological side effect pertains to the development of antibodies against these growth factors, which not only pose risks for future BMP use but may also lead to cross-reactions against naturally occurring growth factors [138,240]. Moreover, the short biological half-lives and localized action of BMP-2 contribute to the associated drawbacks [235]. Thus, the responsible clinical use of BMPs will necessitate further research in developing more sophisticated carriers with biologically suitable release characteristics for growth factors, enabling dose reduction and a more controlled bone formation process [234].

Recently, a promising alternative growth factor has emerged, displaying favorable potential for bone regeneration while avoiding the adverse side effects commonly associated with BMP-2 [144,241,242,243,244,245,246]. Adenosine, known as a protective metabolite, has attracted attention for its osteogenic properties. Traditionally recognized as a cellular-level metabolic marker, adenosine attenuates activity across various cell types as a protective mechanism [245]. However, alterations in bone homeostasis via adenosine receptor activity occur at concentrations above normal physiological levels [241]. In non-stressed cellular conditions, achieving sufficient extracellular adenosine concentrations to trigger receptor activation remains challenging, even with continuous inhibition of the adenosine deaminase enzyme [247]. Consequently, alternative approaches exploring pharmacological manipulation to activate adenosine receptors have been investigated. This manipulation has demonstrated the potential to attain the requisite adenosine concentrations to influence its receptors without inducing stressful cellular conditions. Consequently, alternative approaches exploring pharmacological manipulation to activate adenosine receptors have been investigated. This manipulation has demonstrated the potential to attain the requisite adenosine concentrations to influence its receptors without inducing stressful cellular conditions.

The primary pharmacological agent of note is Dipyridamole (DIPY), an indirect agonist of the adenosine A_2A_ receptor. DIPY operates through the Type 1 equilibrative nucleoside transporter, ENT1, hindering adenosine reuptake into the cell, leading to its extracellular accumulation [248,249,250]. Recent studies have indicated that DIPY not only enhances osteoblast function but also curtails osteoclast formation [251]. Meanwhile, the safety profile of DIPY has been well established after decades-long clinical use in cardiac stress testing and anti-platelet therapy and has been shown to preserve suture patency without indications of ectopic bone formation [248,249,250]. Three-dimensional-printed bioceramic (β-TCP) scaffolds coated with DIPY (3DPBC-DIPY) in various animal models have previously been shown to be effective in bone regeneration for a range of clinical scenarios, including defects induced in the calvaria, ramus, mandible, and alveolus, suggesting adenosine as a promising therapeutic target for rapid bone formation.

A primary objective of regenerative medicine is to create cellular therapies that are free from side effects and devoid of ethical concerns. The utilization of ESCs and IPCSs in therapeutic settings raises several ethical and safety considerations and poses as significant obstacles in clinical applications due to the potential danger of tumor formation [225,252,253]. Alternatively, studies have examined the use of MSCs in surgical procedures for oral and maxillofacial applications. These studies have shown that MSCs can be effectively used for BTE with improved clinical outcomes [225]. MSCs have also been proven to be a more suitable option due to their enhanced biosafety profile and reduced risk of tumorigenicity [254]. However, the ethical and safety issues related to the use of MSC-based treatment are still being discussed, which highlights the need for long-term follow-up research [252].

## 5. Future Outlook and Challenges

Biomaterials and equipment employed in 3DP scaffolds are continuously evolving, becoming increasingly specialized to meet the demands of regenerative medicine and yielding more promising results. However, as discussed in previous sections, there is room for significant improvement. To achieve even more refined outcomes, it is essential to foster collaboration among professionals from diverse fields, including engineers, healthcare practitioners, experts in artificial intelligence, and biomaterial specialists, among others. Teamwork and knowledge sharing facilitate the identification of existing gaps in techniques and promote discussions on potential solutions. With the advancement of various AM technologies, it becomes imperative to establish clear guidelines and standards for the development of 3D-printed scaffolds and their clinical applications. The results of studies already conducted and those currently in progress will play a crucial role in defining these guidelines and will contribute towards more predictable and personalized medical and dental care.

On the other hand, obtaining regulatory clearance for patient-specific 3D-printed medical equipment is challenging. Consequently, incorporating stem cells, growth factors, and other biologics adds more scrutiny and complexity to the approval procedures. It is therefore crucial for researchers to closely follow the rules and guidelines set forth by the regulatory bodies, as this will establish the specific laws they need to adhere to while synthesizing the scaffold/graft or product. Moreover, acquiring a profound comprehension of these demanding procedures will enable craniomaxillofacial surgeons and dentists to render safer and more effective treatment to their patients and will minimize any potential risks while utilizing 3D-printed devices.

## Figures and Tables

**Table 1:** Bioceramic materials used in BTE and their applications.

Ceramic	Sintering Temperature	Characteristic	Applications	Ref.
Hydroxyapatite Ca_10_(PO_4_)_6_(OH)_2_	1000–1250 °C	Capable of fostering cell growth, possessing excellent biocompatibility and good compression strength	Repair of bone defects	[42,43,44]
β-Tricalcium Phosphate β-Ca_3_(PO_4_)_2_	200–1400 °C	Minimal shrinkage, biodegradability, appropriate porosity reduced cracking and deformation	Hard tissue repair of defects	[45,46,47]
Silicon carbide SiC	1860–1950 °C	High strength and good compressive strength	Light weight structural ceramics	[48,49,50]
Zirconium oxide ZrO_2_	1000–1450 °C	Biocompatibility, chemical stability, and excellent mechanical properties	Bone repair and tissue engineering	[51,52]
Barium titanate BaTiO_3_	900–1200 °C	Biocompatible and good tensile strength	Repair of extensive bone defects	[53,54]
Calcium Silicate Bioceramics	1000 °C	Calcination temperature can influence behavior of cells and bioactive on release	Osteogenic differentiation and promote bone regeneration	[55,56,57]

**Table 3:** Summary of commonly used AM methods to produce ceramic based bone replacement scaffolds, and constructs.

AM Method/ Printing Resolution	Ceramic Slurry/Filament/Ink/ Preparation	Commonly Used Materials	Advantages	Disadvantages	Ref.
Fused Deposition Modelling (FDM)/ 100 µm–1 mm	Filaments are produced through a blend of ceramic powders and thermoplastic polymers for 3D printing of structures.	β-TCP, HA, PCL and PLA	Compatible with other materials, reproducibility, low-cost and ease of operation.	Limited resolution and uneven adhesion between layers.	[146,147]
Stereolithography (SLA)/ 20 µm–100 µm	The printing process involves combining ceramics with a photopolymerizable resin.	HA, β-TCP, alumina, ZrO_2_, and bioactive glasses	Low wastage of ceramic materials, high resolution, and printing speed.	Requirement for photopolymers, and the need for subsequent post-processing steps.	[148,149,150]
Selective Laser Sintering (SLS)/ 20 µm–100 µm	The powder bed is prepared with ceramic particles of equal size to withstand laser power and temperature, ensuring a defect-free construct.	PLLA, PCL, HA, and β-TCP	High resolution, fabrication of complex structures using powder as support, and high mechanical strength of printed constructs.	Demand of materials capable of enduring laser heat, managing scaffold shrinkage, and pre- and post-heating treatments.	[151,152]
Direct Inkjet Writing (DIW)/ 100 µm–1 mm	A homogeneous ceramic slurry is created by blending ceramic materials with polymer binders and viscosifiers into the solutions.	β-TCP, HA, and ZrO_2_	Low cost, scalability, capability for fabrication of complex and larger structures.	High pressure, low resolution, needle clogging.	[153,154,155,156]
Digital Light Processing (DLP)/ 25 µm–100 µm	Ceramic powder with liquid photopolymer is exposed to digital light arrays.	HA, β-TCP, and BaTiO_3_	High resolution, cost-effectiveness, and accuracy of print.	Limited availability of materials, requirement for photo reactivity, and restricted build volume.	[148,157,158]
